# Multivalent cationic pseudopeptide polyplexes as a tool for cancer therapy

**DOI:** 10.18632/oncotarget.21441

**Published:** 2017-09-30

**Authors:** Zoi Diamantopoulou, Maud-Emmanuelle Gilles, Maha Sader, Mélissande Cossutta, Benoit Vallée, Claire Houppe, Damien Habert, Blandine Brissault, Eric Leroy, Federica Maione, Enrico Giraudo, Damien Destouches, Jacques Penelle, José Courty, Ilaria Cascone

**Affiliations:** ^1^ Laboratory of Growth, Reparation and Tissue Regeneration (CRRET), University of Paris Est, ERL-CNRS 9215, 94010 Créteil, France; ^2^ East Paris Institute of Chemistry and Materials Science, CNRS & University Paris-Est, 94320 Thiais, France; ^3^ Department of Oncological Sciences and Laboratory of Transgenic Mouse Models, Institute for Cancer Research and Treatment, University of Torino School of Medicine, I-10060 Candiolo, Torino, Italy

**Keywords:** nanoparticles, antitumour activity, polyplex, nucleolin, pancreatic ductal adenocarcinoma

## Abstract

In this study, a novel anticancer reagent based on polyplexes nanoparticles was developed. These nanoparticles are obtained by mixing negatively charged polyelectrolytes with the antitumour cationically-charged pseudopeptide N6L. Using two *in vivo* experimental tumor pancreatic models based upon PANC-1 and mPDAC cells, we found that the antitumour activity of N6L is significantly raised *via* its incorporation in polyplexed nanoparticles. Study of the mechanism of action using affinity isolation and si-RNA experiments indicated that N6L-polyplexes are internalized through their interaction with nucleolin. In addition, using a very aggressive model of pancreatic cancer in which gemcitabine, a standard of care for this type of cancer, has a weak effect on tumour growth, we observed that N6L-polyplexes administration has a stronger efficacy than gemcitabine. Biodistribution studies carried out in tumour-bearing mice indicated that N6L-polyplexes localises in tumour tissue, in agreement with its antitumour effect. These results support the idea that N6L nanoparticles could develop into a promising strategy for the treatment of cancer, especially hard-to-treat pancreatic cancers.

## INTRODUCTION

Nanomedicine, the implementation of nanotechnology-based principles and materials in the medical area, opens new fields of multidisciplinary investigations that could potentially offer innovative sets of diagnostic and therapeutic tools adaptable to several diseases such as cancer. This strategy opens new avenues for better, more adapted or improved treatments of patients. In drug delivery, nanocarriers have been developed that are conventionally defined as engineered structures of nanometric sizes having the ability to carry and release therapeutic molecules to specific disease-related sites. Over the past few years, polymeric nanostructures from natural or synthetic polymers have been extensively investigated in order to develop an effective cancer drug delivery system, which yields a higher therapeutic index of the drug with lower toxicity towards normal tissues, with a few formulations tested under phase I/II human trials [1]. In cancers indeed, due to the high production of proangiogenic factors, tumour vasculature is abnormal with leaky vessels displaying highly tortuous geometries and hyperpermeabilities [2, 3]. These vascular anomalies result in large-size pores in the tumour vasculature as compared to normal tissues, opening a way for nanoparticles to preferentially accumulate in solid tumours [4], ultimately yielding higher therapeutic index of the drug linked to the nanoparticles, and sometimes providing lower toxicities toward normal cells as well.

Up to now, developed nanoparticles have been designed to release the active drug according to two mechanisms, by progressive physicochemical release of the drug encapsulated in the nanoparticle, or as the result of a cleavage reaction involving a covalent bond between the active species and an inert polymer included in the particle, namely the polymer prodrug approach [5]. An alternate design could also be considered, if a particle can be built whose surface is decorated by multiple copies of a substructure acting as a ligand according to a multivalent drug approach. N6L is an anticancer drug developed by our group whose oligomeric structure is made of identical tripeptide repeat units (KΨPR) and whose activity is known to depend upon the number of these units [6]. In this context, the hexamer named N6L, currently investigated under phase IIa trial for cancer therapy, corresponds to a compromise between improved activities, mounting purification difficulties and higher production cost. The above multivalent pseudopeptides target cell surface nucleolin (NCL) and induces apoptosis of tumour cells [6, 7]. N6L decreases the tumour growth of pancreatic ductal adenocarcinoma (PDAC) and reprograms the angiogenesis process by inhibiting angiopoietin-2 [8]. In addition to its effect on tumour growth, N6L also inhibits the migration and invasion of melanoma and PDAC cells [9]. Moreover, high affinity binding of N6L was demonstrated for sulfated glycosaminoglycans (GAG), as well as a displacement of GAG-bound tissue inhibitor of metalloproteinases-3, emphasizing that N6L binds to sulfated GAG [10].

In the present study, we aimed at testing whether higher multivalencies of N6L can be obtained *via* a simple polyplex-based synthesis of nanoparticles acting as scaffolds to display even larger number of active ligands at their surface. An alternative approach to drug-delivery nanoparticles is proposed that explores whether the nanoparticle itself can be used as the active ingredient rather than as a carrier whose sole mission is to transport the active ingredient and to release it over time.

## RESULTS

### Synthesis of N6L Polyplexes

It is well known that mixing opposite charged polyelectrolytes results in the formation of small particles of nano- to micrometric sizes [11]. These particles, usually referred to as polyplexes, are colloidally stable, and in most cases, do not aggregate due to repulsive force. In biomedicine, these polyplexes have mostly been used as carriers to deliver therapeutic nucleic acids [12]. In a previous study, we reported that N6L, which under physiological pH is a highly-charged oligomer with 24 positive charges distributed over a small volume, shows a high affinity interaction for heparin [6]. In this study, we sought to test whether the interaction of N6L with heparin or other sulfated glycoaminoglycans could lead to the formation of nanostruture such as polyplexes.

To test whether N6L could yield polyplexes with heparin, an aqueous solution containing both N6L and heparin was prepared and added to a dynamic light scattering (DLS) cuvette after mixing to reveal the presence of nanoparticles. Results indicated that a monodisperse size distribution is observed, with an average particle diameter of 194 ± 3 nm at final concentrations of 14.7 μM and 100 μg/mL in N6L and heparin, respectively (Supplementary Figure 2). An additional measurement after 24 h at room temperature led to an equally monodisperse nanoparticle, with a diameter of 193 ± 10 nm (not shown).

**Figure 1:**
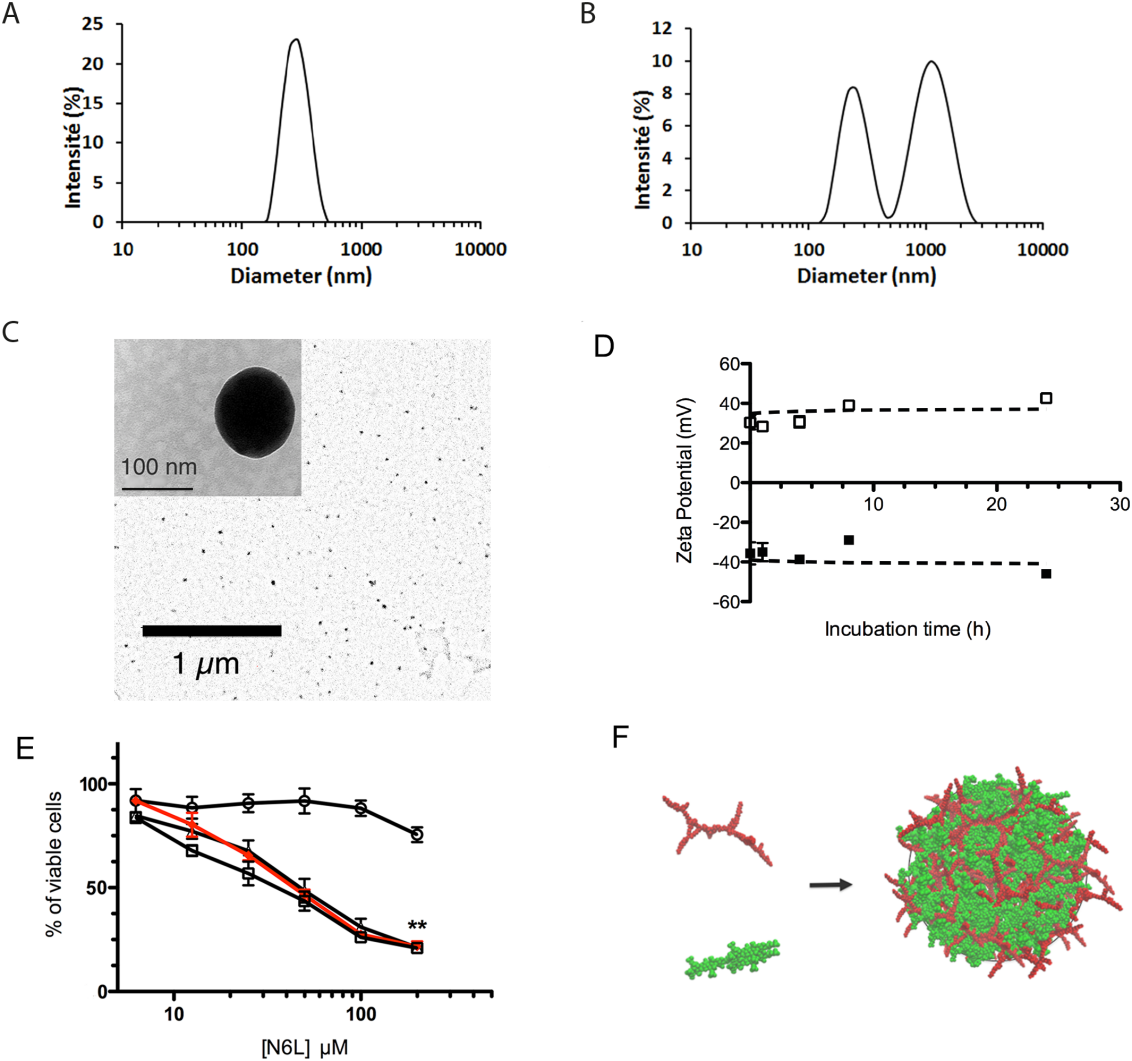
N6L and anionically charged polyelectrolytes spontaneously assemble into functional polyplexed nanoparticles **(A** and **B)** DLS measurement of particle size distribution measured after mixing chondroitine sulphate (CS-C) with N6L in water at 23°C (final concentrations for CS-C of 10 μg/mL (A) or 1000 μg/mL (B), and 73 μM for N6L in both cases). These data are representative results obtained from three consecutive measurements of 15 runs each; **(C)** typical scanning transmission electron microscope (TEM) image of polyplexed particles obtained after mixing 10 μg/mL CS-C with 73 μM N6L in water; **(D)** Zeta potential determination of N6L polyplexes: the charge of the polyplexes obtained with 73 μM N6L and 1000 μg/ml of CS-C (black square) or with 73 μM N6L and 10 μg/mL CS-C (open square) were measured as a function of the incubation time ranging from 0 to 24 hours. Data smoothing using the GraphPad software indicates that potentials resulting from polyplexed N6L (73 μM N6L) were stable over a period of 24 hours, with value of 34.2 ± 6.5 mV and -38.1 ± 7.3 mV for CS-C 1000 and 10 μg/mL, respectively; **(E)** PANC-1 cells were treated with concentration of N6L ranging from 4.6 μM to 147 μM, alone or polyplexed by adding CS-C at concentrations of 1000 (black circle), 100 (black triangle) and 10 μg/mL (black square). After 72 hours, cell growth was quantified by AlamarBlue assay according to the recommended procedure. Histograms represent the percentage of cell growth relative to the values of untreated cells (indicated values are means ± SEM of 3 independent experiments (Student *t*-test, n=3)); **(F)** schematic illustration for the obtained polyplexed structure (red: N6L building blocks, green: CS-C octamer used as a model for fully developed CS-C).

To validate these data, similar experiments were performed using another sulfated glycosaminoglycan, chondroitin sulphate C (CS-C), as the polyanionic macromolecule, at 10 or 1000 μg/mL and 73 μM for CS-C and N6L, respectively. A monodisperse hydrodynamic size distribution was observed with an average diameter of 279 ± 11 nm, at concentrations of 10 μg/mL and 73 μM in CS-C and N6L, respectively (Figure 1A). When higher concentrations of CS-C (1000 μg/mL) were used, relatively large and polymodal size distributions were observed, with average diameters at 228 ± 25 and 973 ± 187 nm for the two major populations (Figure 1B). Confirming the data presented in Figure 1A, we observed by transmission electronic microscopy (TEM) that nanoparticles obtained by mixing a solution of 10 μg/ml CS-C and 73 μM N6L have a particles diameter of about 100 nm (Figure 1C). We next studied the stability of these N6L-polyplexes over 24 hours periods by measuring the change in particle hydrodynamic size. As shown in Table 1, while N6L-polyplexes obtained with 1000 μg/mL CS-C remained relatively stable over a period of 4 hours, a significant size increase of N6L-polyplexes obtained with 10 μg/mL CS-C could be observed 8 hours after the polyplex formation, reaching a diameter of 381 ± 88 nm. Determination of the zeta potential indicated that polyplexed N6L obtained from 73 μM N6L mixed with 10 and 1000 μg/mL of CS-C display values of +34.2 ± 6.5 mV and -38.1 ± 7.3 mV, respectively, numbers that remained stable over at least 24 h (Figure 1D).

**Table 1:** Evolution of polyplex sizes as a function of time

Polyplex CS/N6L (mg/μM)	Time (h)	Size 1 (nm)	Size 2 (nm)
1000/73	0^*^	187 ± 15	1190 ± 420
	1	245 ± 85	1150 ± 245
	4	228 ± 25	973 ± 187
	8	nv	nv
	24	nv	nv
10/73	0^*^	nv	nv
	1	nd	-
	4	279 ± 11	-
	8	460 ± 24	56 ± 37
	24	616 ± 88	97 ± 1

The sizes of the particles were determined by DLS as described in Materials and Methods. nv: not validated; nd: not determined. (^*^) Size determination was measured 15 minutes after having mixed N6L and CS-C.

To examine the potential anticancer activity of N6L-polyplexes, we tested its effect on the viability of the well-established human pancreatic cancer cell line PANC-1. Cells were treated with several concentrations of N6L ranging from 4.6 to 147 μM, alone or in the presence of several concentrations of CS-C. As shown in Figure 1E, as compared to the treatment of cells with N6L alone, N6L-polyplexes obtained at CS-C concentrations lower than 1000 μg/mL (*i.e.*, at 100 and 10 μg/mL) displayed a similar effect to those observed for N6L used alone (Figure 1E). No effects on the cell viability could be observed whatever the concentration of CS-C used in the experiment (Supplementary Figure 3).

Taken together, all these observations provide evidences that mixing N6L with sulfated glycoaminoglycans like CS-C induces the formation of polyplexed nanoparticles, according to a global aggregation mechanism schematized in Figure 1F. For the rest of the study, we decided to investigate the properties of N6L-polyplexes obtained by mixing N6L with 10 μg/mL of CS-C.

### Molecular functionality of N6L-polyplexes

**Figure 2:**
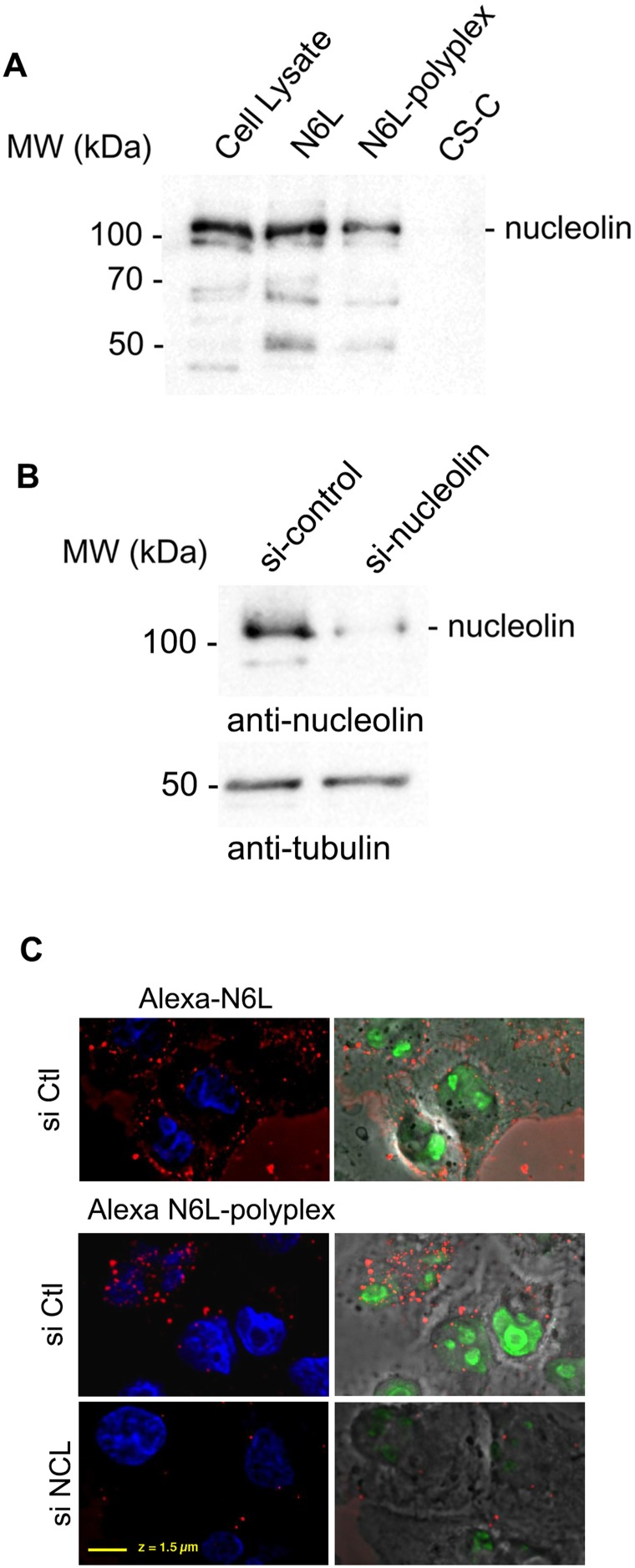
Biological properties of N6L-polyplexes **(A)** Polyplexed N6L interacts with nucleolin. PANC-1 cells were incubated (lane N6L and N6L-polyplex) with 5 μM of biotin-labeled N6L in soluble (lane N6L) or polyplexed form (N6L-polyplex) or CS-C as a control (lane CS-C) for 1 h. N6L-interacting proteins were isolated by incubating cell lysates with avidin-agarose beads. Samples were analysed by SDS-PAGE, and Western blotting performed by using an anti-nucleolin antibody. **(B** and **C)** PANC-1 cells pre-treated (or not) with nucleolin siRNA (siNCL N6L-polyplex), GFP siRNA (siCtl – N6L-polyplex), as described in Materials and Methods, were incubated for 10 min with 2 μM of polyplexed N6L-Alexa fluor 588. Cells were then fixed in paraformaldehyde, stained with diamidino-2-phenylindole (DAPI) (Molecular Probes), and analysed. The efficiency of si-nucleolin is shown in the figure by immunohistology using anti-nucleolin antibody and (B) by Western blot analysis.

Previous studies have shown that N6L interacts with nucleolin [6]. To address whether the inhibitory effect of the N6L-polyplexes is linked to their interaction to nucleolin, we performed streptavidin pull-down assays using PANC-1 cell lysates incubated with biotin-labelled N6L-polyplexes. As shown in Figure 2A, N6L-polyplexes interact with nucleolin in a similar way to N6L alone, suggesting that nucleolin might be responsible for the anti-tumoural effect of the N6L-polyplexes.

According to our previous study in which we clearly showed that the mechanism of action of N6L occurs through its internalization in target cells [6], we next investigated the cell internalization of N6L-Alexa fluor 488 in its polyplexed form. As shown in Figure 2B and 2C, 10 minutes after the addition above polyplexes, fluorescence could be detected inside the cells. These data were reinforced by observations carried out when invalidating nucleolin expression. Efficiency of si-nucleolin (siNCL) is shown by Western blot analysis (Figure 2B). As evidenced when compared to a control (SiCtrl alexa N6L-polyplex), inhibition of nucleolin expression (siNCL alexa N6L-polyplex) prevented N6L-polyplex internalisation (Figure 2C). Taken together, these data indicated that N6L-polyplex is rapidly internalized into the cell through nucleolin.

### *In vivo* anti-tumoural and anti-metastatic effect of N6L polyplexes

**Figure 3:**
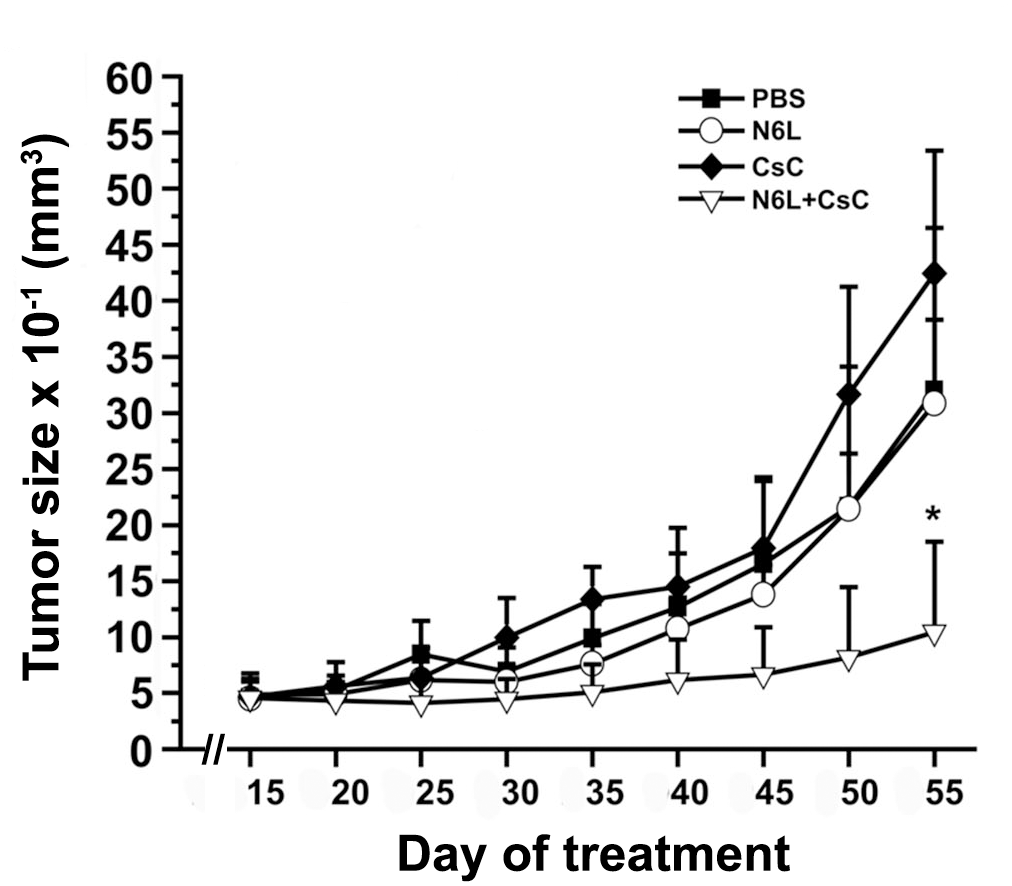
N6L polyplexes inhibit tumour growth of N6L-resistant tumours Athymic mice were subcateneously injected with PANC-1 cells. Ten days after cell injection, mice were treated with intraperitoneal injections of either N6L (2 mg/kg of body weight) or N6L polyplexes (2 mg/kg, ratio CS-C/N6L of 1/8 w/w), CS-C (0.26 mg/kg of body weight), or PBS as control, three times per week. The tumour volume was measured as described in Materials and Methods. Bars ± SEM (n = 9 per group).

**Figure 4:**
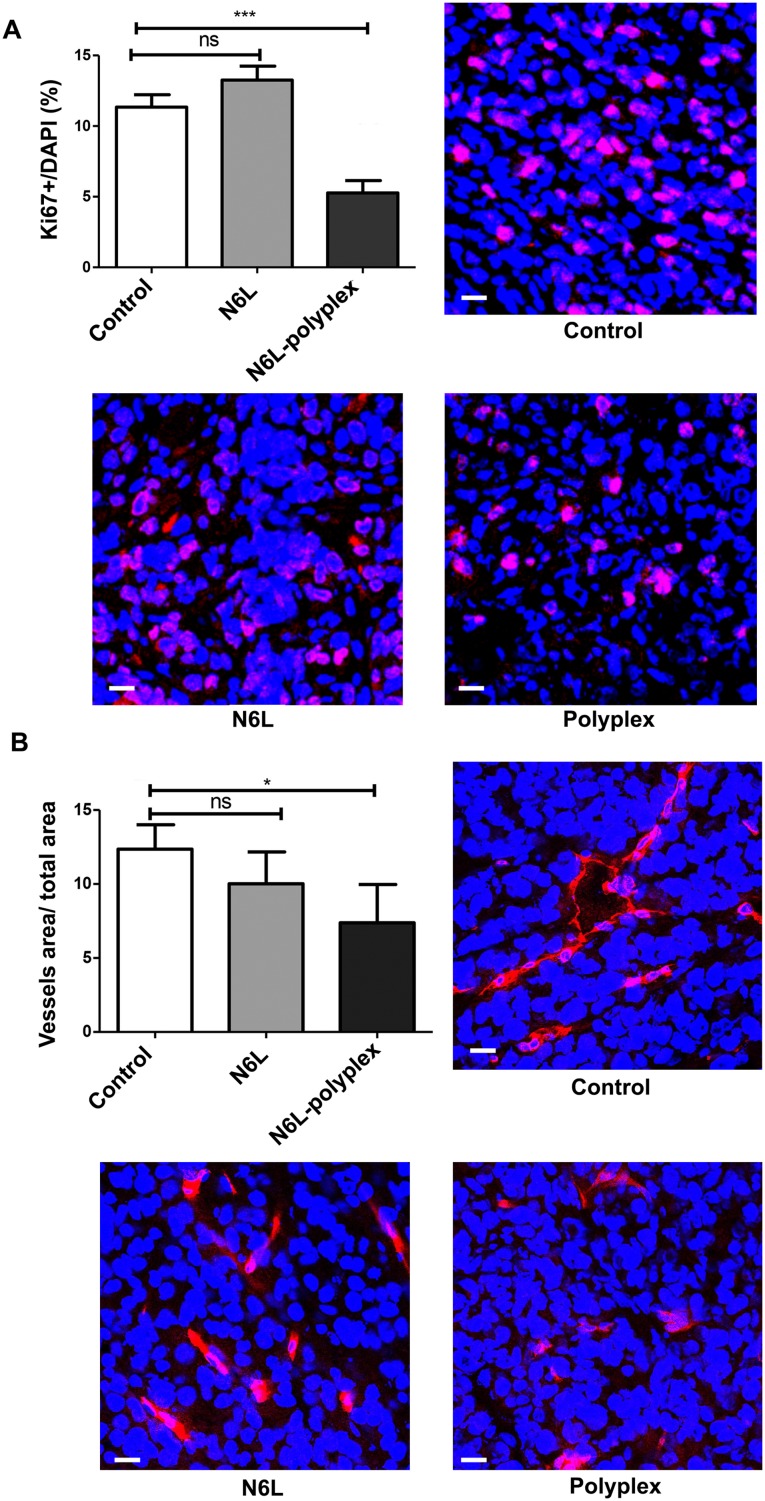
Effect of N6L-polyplexes on tumour proliferation and vascularisation Frozen tumour sections were fixed, saturated with BSA 3% for 1 h at room temperature, and incubated with anti-ki67 **(A)** or anti-MECA32 **(B)**. After washing, sections were incubated with anti IgG Alexa Fluor-555. Nuclei were stained with DAPI, and immunofluorescence images were captured by confocal microscopy. Acquisitions were carried out on multiple tissue sections (5 field per mouse, n = 5), and quantification analysis was performed by the ImageJ sofware.

*In vivo* anti-tumour activity of N6L polyplexes was then analysed using subcutaneous tumor xenograft of PANC-1 cells. As shown in Figure 3, treatments with N6L alone had no significant effect on PANC-1 xenograft tumour growth. In sharp contrast, N6L polyplexes significantly inhibited tumour growth by 85% (p<0.05) (Figure 3). No significant effect of CS-C alone could be observed. Immunohistological analysis of tumour indicated that treatment with N6L polyplexes significantly decreased Ki67 positive cells by 53 % (p<0.001) (Figure 4A). Moreover, the area occupied by the tumour vasculature decreased by 70 % (p<0.001) (Figure 4B).

All animals were closely monitored during the treatment and no toxicity could be evidenced in the various groups involved in the experiments, including the group treated with the N6L polyplexes (data not shown).

**Figure 5:**
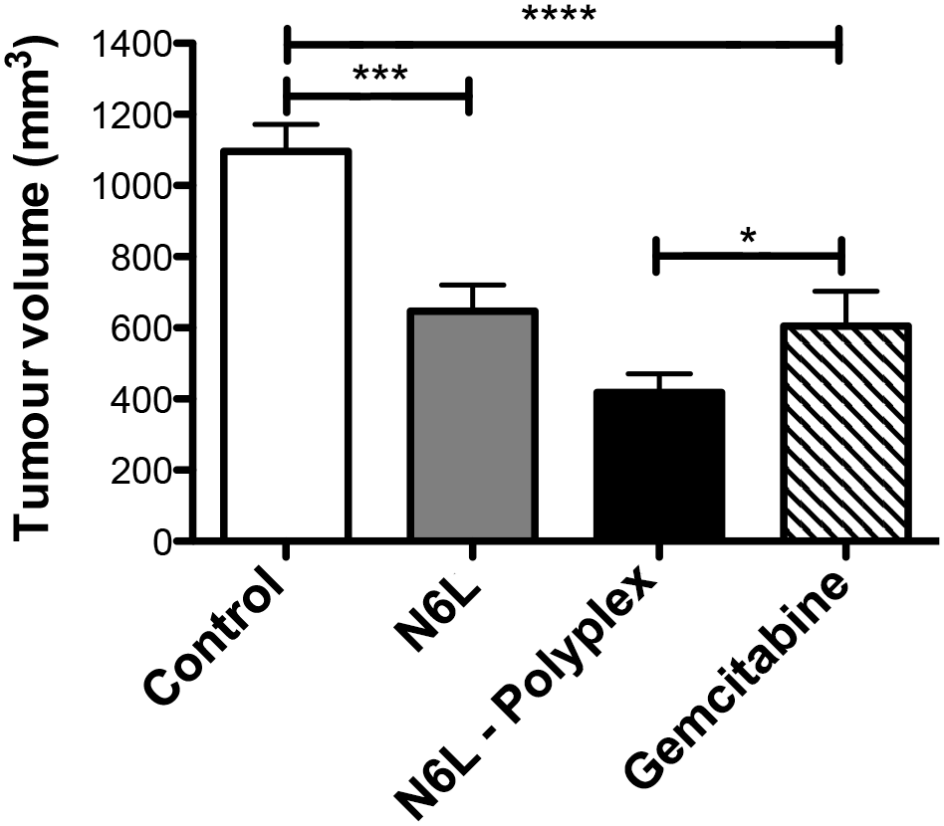
N6L polyplexes reduce PDAC tumour growth Immuno-competent syngenic FVB/n mices were injected with PDAC cells into the pancreas. One week later, either N6L alone (10 mg/kg) or N6L polyplexes (10 mg/kg) were injected (i.p.) three times a week. Control were carried out by injection of gemcitabine (100 mg/kg) two times a week. After three weeks of treatment, mice were sacrificed, and tumour volumes were measured (n = 10, student *t*-test, ^***^ P < 0.001; ^*^ P < 0.05).

We then evaluated the antitumour effect of N6L polyplexes in the mPDAC orthotopic model in which N6L inhibited tumor growth [8]. After 3 weeks of treatment, N6L decreased the tumour volume by 42 % (p < 0.001), a value close to the literature value obtained when using gemcitabine (45 % (p < 0.01)) [8]. Interestingly, N6L polyplexes displayed a greater antitumour activity compared to gemcitabine by significantly reducing tumour burden by 72 % (Figure 5). A treatment with CS used as a single agent did not exert significant inhibitory effect on PDAC tumours (not shown).

Collectively, these results demonstrated that N6L polyplexes have a much stronger antitumoural effect than N6L used alone.

### Biodistribution of N6L and N6L-polyplex

**Figure 6:**
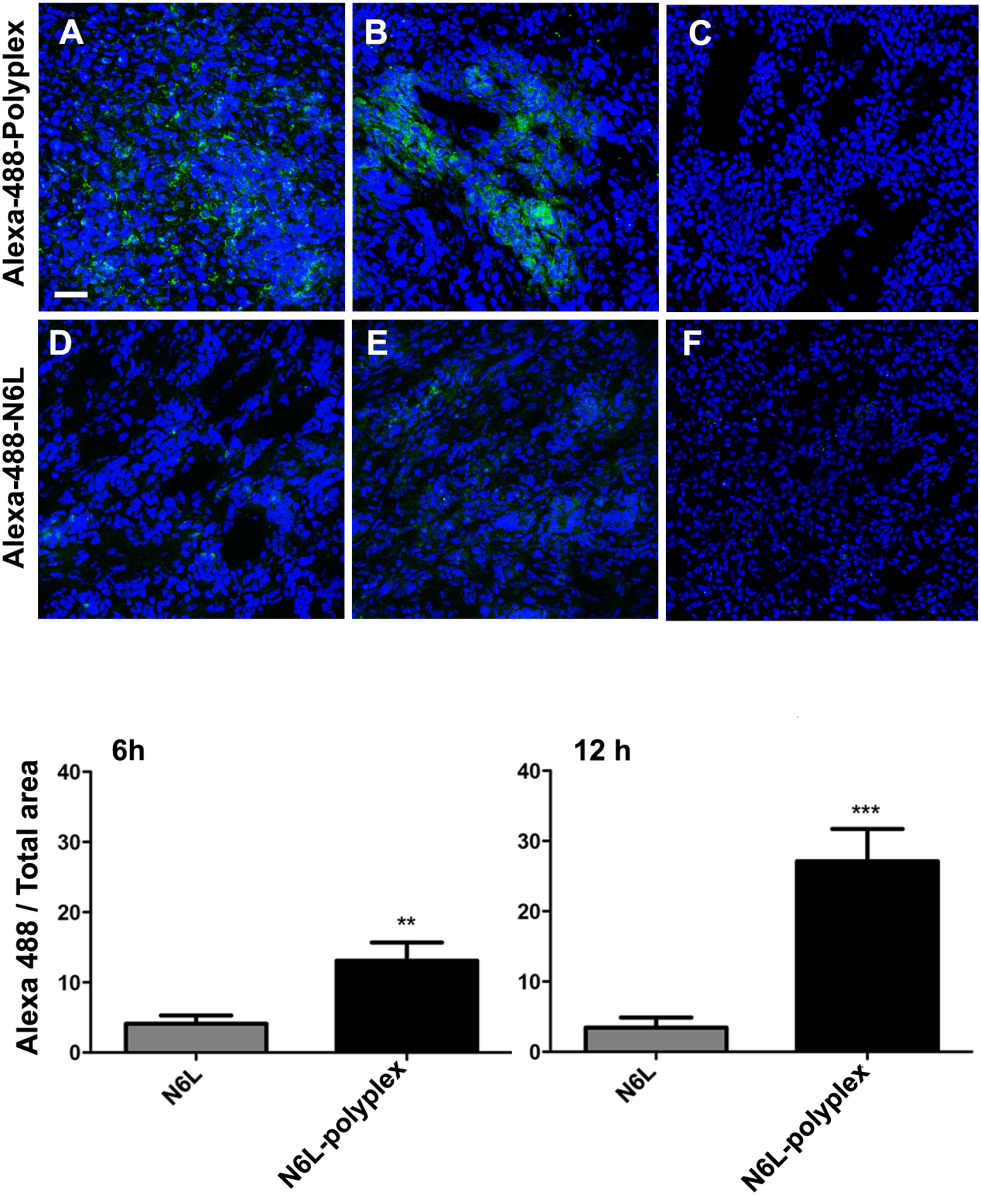
N6L polyplexes target tumour tissue **(A)** N6L-polyplex targets pancreatic tumour tissues more efficiently than N6L alone. Mice with established PDAC tumours (A, **B, D and E)** or mice without tumours **(C** and **F)** were injected with Alexa-488-stained N6L, alone or in a polyplexed form, and sacrificed after 6 hours (A and D) or 12 hours (B, C, E and F). Tumours were recovered, and confocal analysis was performed on tissue sections. Images are representative of 5 fields observed. N6L fluorescence intensity was quantified and plotted (n = 5 at least; student *t*-test, treated *vs.* not-treated, ^***^ P < 0.001, ^**^ P < 0.01). Alexa-488 N6L was not detected in normal pancreas (C and F).

We sought to correlate the effects of N6L polyplexes with the ability to be delivered to the tumour in the mPDAC model. In order to reach this objective, we performed comparative tissues distribution studies using injections of fluorescent N6L polyplexes and fluorescent N6L alone. Pancreas cancer tissues from mPDAC mice were examined 6 and 12 hours after intravenous injection of either polyplexed N6L-alexa 488 or N6L-alexa 488 used alone. A strong fluorescent signal was observed in sections of pancreatic cancer tissues of mice injected with the polyplexed N6L-alexa 488 (Figure 6A and 6B). Fluorescence could be observed starting from 6 hours after the injection of N6L polyplexes (Figure 6A) to 12 hours (Figure 6B). The fluorescent signal was much higher in tumours treated by N6L polyplexes than N6L alone (Figure 6D and 6E). Whatever the molecular form of N6L-alexa fluor 488 injected, it is noteworthy that no fluorescence could be observed in sections of normal pancreatic tissue (Figure 6C and 6F).

## DISCUSSION

Polyplexed nanoparticles have been already used in cancer therapies, not only in research but also increasingly in clinical trials. However, most of these studies are based on the use of polyplexes made from DNA and polycations of different natures in the perspectives of non-viral delivery systems for cancer gene therapy. Due mainly to their instability, few studies have been reported on the therapeutical use of polyplexes other than the DNA-based ones. In this study, a stable polyplex nanoparticles with antitumour activities were obtained through self-assembly between N6L and sulfated glycosaminoglycans used as anionic polymers.

From a physic-chemical perspective, polyplexes are particles of small sizes suspended in water that form as the result of strong attractive electrostatic interactions between charged polymers of opposite charges in aqueous solutions [13]. Their sizes, typically in the nanometric range, depends very much on both the nature of the involved polyelectrolytes and the mixing conditions (e.g., the molar ratio between the partners, their initial concentrations, the presence of other species or the temperature) [14, 15]. As indicated above, DNA/RNA-based polyplexes with various positively charged polyelectrolytes have been particularly investigated in the past few decades as a potential non-viral delivery method for gene therapy [16]. As heparin is characterized by a highly negatively charged polysaccharide backbone comprised of sulfated uronic acid-(1→4)-D-glucosamine repeat units, and N6L displays 24 positive charges at physiological pH, it is tempting to assume polyplexes would be formed when mixing cationically-charged heparin and negatively-charged N6L together.

Investigated polyplexes were formed according to two sets of preparation conditions (i.e., in water and in a cell culture medium) that had been selected to mimic those used in the *in vitro* experiments. Unfortunately, it was impossible to obtain dependable results in a cell culture medium. The high concentration of proteins in such media generates indeed a large scattering signal, the high number of small-size light-scatterers compensating for their much lower scattering power with respect to the one generated by much larger polyplex particles. This background scattering induces a major difficulty when attempts are made to mathematically generate a unique, statistically robust solution from the autocorrelation function from a single-angle scattering measurement [17, 18].

From these experiments, the conclusion could be reached that CS-C undeniably induces the easy formation of polyplexes when mixed with N6L, whatever the preparation conditions. In addition, the above experiments supported the assumption that the bulk of the bioactive N6L ingredient used in the upcoming *in vivo* and *in vitro* experiments should be located in the aggregated polyplexed particles, at least initially.

A remarkable feature of the results presented in this study is that a polyplexed structure of N6L improved drastically the antitumour response of N6L compared to N6L used alone. This effect was demonstrated using several tumour growth models of human pancreatic tumours, using heterotopic and orthotopic grafting experiments. Pancreatic cancer had been selected because they are very difficult to cure and, despite the intensive research efforts that have been developed so far, remains resistant to the majority of cytotoxic drug currently used as well as to conventional radiochemotherapeutical treatment [19]. It is noteworthy that, in the above models, the therapeutic effect of polyplexed N6L is better that those obtained with a sub-toxic dose of gemcitabine, which is the current reference treatment for this type of cancer. In addition and as previously described with N6L used alone, no toxicity of the polyplexed N6L could be observed when used at therapeutic doses. As previously described for N6L, the antitumour activity of N6L-polyplex is not specific for pancreatic cancer because antitumour effect of N6L-polyplex is also observed using U87 cells as target cells [20].

**Figure 7:**
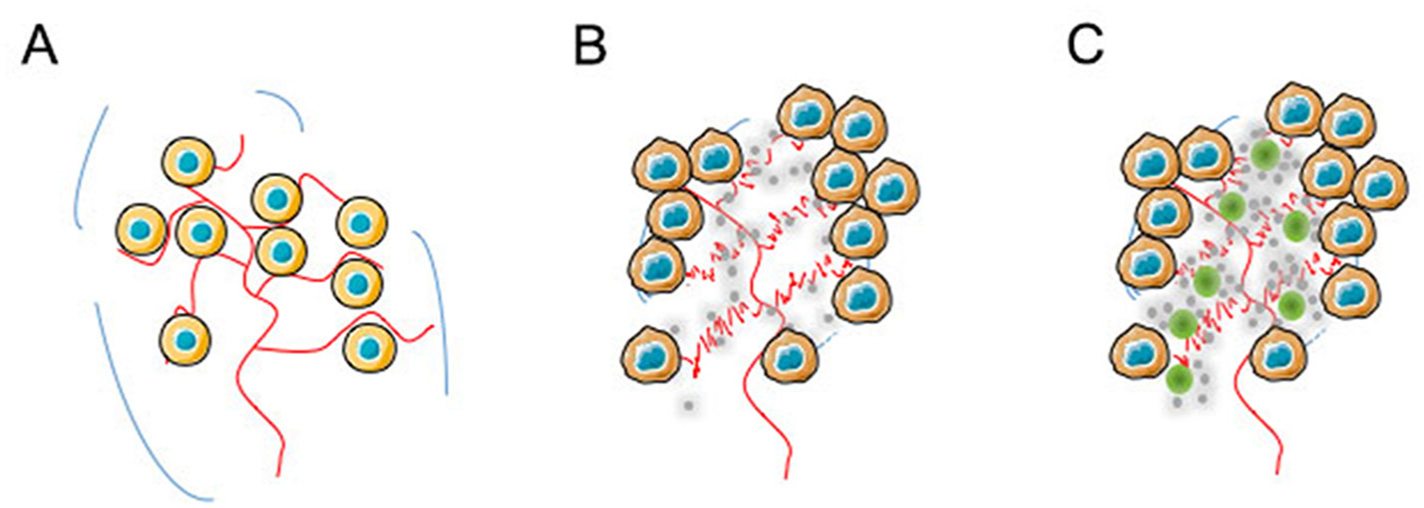
Illustration of a possible mechanism for tumour bioavailability based on N6L-polyplexes In the light of our accumulated data, the enhancement in antitumour efficacy for N6L in a polyplexed form compared to N6L used alone could be explained by EPR effect. In normal pancreas **(A)** in which lymphatic clearance occurs, and nucleolin expression at the cell surface of endothelial cells from normal vessel does not whatever the N6L formulation injected, no retention of N6L in the tissue could be observed (see Figure 6C and 6F). **(B)** In contrast, administration of N6L alone (grey circles) to animals bearing pancreatic tumour localizes into tumour tissues (Figure 6E and 7B) in which high expression of nucleolin had been observed [8]. **(C)** When N6L-polyplexes were administered (green and grey circles), it can be assumed that N6L nanoparticles are concentrated by the EPR effect to the tumour microenvironment, increasing N6L bioavailabilty for the tumour as shown in Figure 6A and 6B.

It is now well-established that, under certain pathophysiological circumstances like tumour development, tumour blood vessels become more permeable than under normal physiological conditions [21], resulting in their barriers becoming leaky. Under such conditions, large molecules and particles until 500 nm in size can leave the vessel and accumulate inside the intersticial tissue of the tumour [22]. Among the various parameters modulating this effect, the most important appears to have biocompatible molecules displaying molecular weights larger than 40 kDa. In the light of our accumulated data, the enhancement in antitumour efficacy for N6L in a polyplexed form compared to N6L used alone could be rationalized according to the so-called EPR (Enhanced Permeability and Retention) effect, which a model is presented in Figure 7. Hence, whatever the N6L formulation injected to the mice, no N6L retention could be observed in normal pancreas tissues (Figure 7A), where lymphatic clearance and the absence of nucleolin expression at endothelial cell surfaces in normal vessels simultaneously occurred. Interestingly, as decribed for mice bearing MDA-MB 231 tumours [6] in *in vivo* imaging experiments, administration of N6L to animal bearing pancreatic tumours induced N6L localization in the tumour tissue (Figure 7B) where high mucleolin expression has been observed [8]. In the case of N6L-polyplex administration, it can be assumed that N6L nanoparticles are concentrated in the tumour according to the EPR effect, increasing its bioavailabilty (Figure 7C) as compared to an administration of N6L alone. Under these conditions, we can assume that N6L diffuses from the nanoparticles to the tumour environment. This possibility is consistent with the observation that polyplexed N6L can cross a dialysis membrane with a molecular cut-off of 8,000 Da according to the low of mass action (Supplementary Figure 4). Taken together, these data suggest that N6L polyplexes could be considered as a reservoir system for N6L. Surprisingly, although N6L binds to sulfated glycoaminoglycans with high affinity, N6L polyplexed with CS or heparin (not shown), retained its ability to inhibit the tumour cell proliferation and to induce apoptosis. However, it is noteworthy, that the biological effect of polyplexed N6L is a function of the CS concentration used to form the nanoparticles. As presented in this study, high concentration of CS inhibited the biological activity of N6L. This observation suggests that the functional domains in polyplexed N6L are constituted by the positive charges of KPR domains in N6L, which are neutralized by the negative charges in CS. This hypothesis is conforted by the fact that the specific activity of the multivalent pseudopeptide N6L is dependent upon the number of KPR tripeptides present as lateral branches (see Supplementary Figure 1) [6].

Sulfated glycoaminoglycans are natural polysaccharides present in mammalian tissues. They contribute to critical functions in the physiological and pathological mechanisms involved in maintaining homeostasis [23]. Interestingly, these polysaccharides have already been used as pharmacological agents in cancer therapies. Even if our models do not suggest any antitumour effect of CS alone, it cannot be excluded that the use of other natural or synthetic polyanionic molecules displaying both a high affinity for N6L and antitumour activities could not induce better results than those obtained with CS. In this context, it can be noted that modified CS like neoglycans induce apoptosis in myeloma cells, [24] and reduce or even fully prevent the growth of breast cancer in a xenograft model without apparent toxic effect. Also, highly sulfated polysaccharides isolated from sea cucumber display antimetastatic activity through the binding to P-selectin [25]. Moreover, it has been shown that acharan sulfate, a glycoaminoglycan isolated from the giant African snail, inhibits tumour growth in Lewis lung carcinoma without toxicity or resistance effects [26]. Other studies have indicated that a treatment of human bladder cancer cells with a combination of CS and either gemcitabine or mitomycin-C results in a marked synergic antitumoural effect [27]. These data suggest that it could be very interesting to use polyplexed N6L as a nanoparticles platform for synergistic chemotherapeutic drugs delivery. This possibility is currently under investigation using both natural and synthetic polyanionic molecules. Another advantage of the N6L polyplexes shown in this study is the ability of these nanopaticules to target tumour cells after their administration through the blood stream. This property has been already observed using other molecules targeting cell surface nucleolin such as AS1411 aptamer [28] and the F3 peptide [29], validating this molecular target. Recently, these molecules have been used to develop a nanoplatform to codeliver several anticancer drugs and improve the delivery of these molecules to cancer cells [30–33]. In previous studies, we have developed iron oxide nanoparticles functionalized by the multivalent pseudopeptide N6L that target breast tumor by binding to nucleolin and sulfate glycoaminoglycans. However, due mainly to the amount of N6L linked to the magnetic nanoparticles, although these nanoparticles target tumor, no effect on the viability of tumor cells have been observed [34]. From these studies, it is noteworthy that the combination of magnetic nanoparticles with sulfate glycoaminoglycans as a binding site for N6L will be interesting both for tumor targeting properties as well as antitumor activity. In addition, as previously mentioned [35], using N6L-polyplex approach combining magnetic hyperthermia and chemotherapy will represent an interesting therapeutic strategy for cancer treatment.

In conclusion, the development of theragnostic, which is a strategy that combines therapeutics with diagnostics constitutes a promising approach that we currently develop with N6L-polyplexes [34].

## MATERIALS AND METHODS

### Preparation of peptide constructs and N6L-polyplexes

The synthesis of N6L was performed as previously described [6]. A fluorescent conjugate and biotin conjugate were prepared from the N6L-Cys derivative as previously described [6]. The conjugates were purified by high-performance liquid chromatography and lyophilized. Polyplexed N6L nanoparticles aimed at biological testing were produced before each experiment by mixing equal volumes of 4 mM N6L and 20 mg/mL heparin (Sigma-Aldrich, St Quentin Fallavien, France) or chondroitin sulfate C (Cs-C) (Baccinex, Courroux, Switzerland) solutions. After 15 minutes, an opalescent solution could be observed. Nanoparticles were diluted at the concentrations indicated in the figure captions.

### Design of N6L polyplexes for structural characterization

Dynamic light scattering measurements were obtained at a 173° angle with a Nano-ZS Zetasizer from the Malvern company, equipped with a 4 mW He-Ne laser at 633 nm. Measurements were obtained in triplicate (each measurement was the average of fifteen 10 seconds runs). The data were analyzed using Malvern Zetasizer Series Software v7.11. A Malvern Instruments dip cell was used for zeta potential measurements, each sample being measured in automatic mode, with an imposed maximum of 100 runs. For the DLS experiments, samples were prepared as follows. Stock solutions of N6L (2.8 mM in water), of heparin (19.6 mg/mL in water), and of Cs-C under three conditions (20 mg/mL in water, 1 mg/mL in water, and 1 mg/mL in a culture medium) were prepared. In the case of heparin, polyplexes were produced by mixing equal volumes of N6L and heparin solutions. After 15 minutes, the obtained mixture was diluted 100-fold in water. In the case of Cs-C, polyplexes were produced by mixing equal volumes of N6L and Cs-C (20 mg/mL in water solutions). After 15 minutes, the mixtures were diluted by a ten-fold factor, either in water or in the above culture medium, as indicated in the text or in figure captions. The obtained diluted solutions were further diluted by a two-fold factor, using either one of the following Cs-C stock solutions (i.e., 1 mg/mL in water or 1 mg/mL in the culture medium), as indicated in the text or in figure captions.

Transmission Electron Microscopy (TEM) images were obtained using a FEI Tecnai F20 microscope operating at an acceleration voltage of 200 kV. Bright Field images were recorded using a Gatan Orius 1000 CCD camera. The experiments were carried out at room temperature. Samples were prepared as follows. A drop of the polyplex solution was applied on a 400 mesh copper grid covered by a Formvar/C membrane, which had previously been treated by air plasma to make the surface hydrophilic. After 1 minute, the excess liquid was removed with adsorbent paper and stained with a 0.7 % (w/w) water solution of uranyl acetate. The grid was subsequently dried in air at room temperature.

### Cell culture and treatments

Human pancreatic carcinoma cell line (PANC-1; ATCC® CRL-1469™) was obtained from the American Type Culture Collection (ATCC) and cultured in DMEM 10% FCS. For viability experiments, 1.5 × 10^5^ PANC-1 cells per cm^2^ were plated at day 0 on 48-wells cell culture plate (Falcon). At 24 h, cells were treated by the investigated molecules in DMEM 5% FCS. Quantification of cells was performed using the blue Alamar assay (Sigma-Aldrich, St Quentin Fallavien, France). Small interfering RNA transfection was performed using RNAiMAX kit (InVitrogen) and 20 nM of nucleolin siRNA (sequence: CCACAAGGAAAGAAGACGAAG, Abnova corporation, France) or GFP siRNA, which was used as nontargeting control siRNA (Ambion, Thermofisher Scientific). Efficiency of si-nucleolin was controled by Western blot analysis and by immunohistology as previously described [8]. Mouse pancreatic ductal adenocarcinoma (mPDAC) were kindly provided by Douglas Hanahan's group (ISREC of Lausanne, Switzerland) [36], and cultivated as previously described [8].

### N6L-fluorescent conjugate uptake and immunofluorescence

PANC-1 cells were plated at 80% of confluence on slides in 24 wells, and treated with 2 μM of N6L-alexa fluor 588 alone or in a polyplexed form for 10 minutes at 37°C. Cells were fixed in paraformaldehyde 4% for 10 minutes at room temperature, and cell nuclei were stained with diamidino-2-phenylindole (DAPI) (Molecular Probes). Cells were analyzed by using a Leica SPEII confocal laser-scanning microscope (Leica Microsystems).

### Affinity isolation experiments

Affinity isolation experiments were performed as previously described [6]. Briefly, PANC-1 cells were plated on dishes (Falcon), and grown to at least 80% cells confluence. Biotin-labeled N6L (5 μM, alone or in a polyplexed form) was diluted in DMEM supplemented with 5 % FCS and incubated with cells for 1 hour at 37°C and 5% CO_2_. After washing the cells once in PBS, cell extracts were prepared in lysis buffer containing 20 mM Tris-HCl (pH 7.5, 50 mM NaCl, 5 mM MgCl_2_), 1 μl/mL protease inhibitors obtained from Sigma-Aldrich, 0.5% Triton X100, 1 mM NaF, and 1 mM Na_3_VO_4_. The complexes formed between biotin-labeled N6L and interacting proteins were isolated by purification of the extracts, using 100 μL avidin-agarose (Pierce, Thermo Fisher Scientific) in PBS containing 1 mM EDTA, 1 mM NaF, and 1 mM Na_3_VO_4_. After overnight incubation at 4°C, the samples were washed extensively with the same buffer. The purified proteins were subjected to SDS-PAGE and Western blot analysis using anti nucleolin antibody (rabbit polyclonal, Abcam ab22758).

### *In vivo* experiments

Experiments with human solid tumour xenografts in nude mice were carried out as previously described [9]. Athymic 4-week-old Nu-NU mice (Janvier Labs, Le Genest St Isle), were subcateneously injected with either 5 × 10^6^ PANC-1 cells or 1.5 × 10^6^ U87-MG cells. When the tumour reached about 40 mm^3^, the mice were separated randomly in several groups, then treated with an intraperitoneal injection of either N6L (2 mg/kg of body weight) or N6L polyplexes (2 mg/kg, ratio CS-C/N6L of 1/8 w/w), CS-C (0.26 mg/kg of body weight) or PBS as control, three times per week. The tumour volume was measured along two major axes with calipers. Tumour volumes (mm^3^) were calculated as follows: V = 4/3×π×R_1_^2^×R_2_, where R_1_ is radius 1, R_2_ is radius 2 and R_1_<R_2_. The mouse model of pancreatic ductal adenocarcinoma was obtained by injecting orthotopically in a cohort of FVB/n syngenic mice clonal tumour lines (0.5 × 10^3^ cells/mouse) isolated from the p48-Cre, LSL-KRasG12D; p53flox/wt and the p48-Cre, LSL-KRasG12D; Ink4a/Arfflox/wt transgenic PDAC models. These cells are mutated for KRas and p53 and are null for Ink4a/Arf. When injected into the pancreas of immuno-competent FVB/n mice, these lines were able to form tumours that recapitulated many feature of the spontaneous tumour microenvironment with an average latency of 3-4 weeks. One week after cell inoculation, PDAC mice were treated 3 times/week for a total of 3 weeks with an intraperitoneal injection of either N6L (10 mg/kg) or N6L polyplexes (10 mg/kg, ratio CS-C/N6L of 1/8 w/w) or vehicle (saline solution) as control. Pancreas from mice were dissected and collected. The total tumour burden was quantified by measuring with a caliper and estimating the volume of individually excised macroscopic tumours (>1 mm^3^) with the formula: V= a x b^2^ x 0.52, where a and b represent the longer and shorter diameter of the tumour, respectively. All animal procedures had been approved by the Ethical Commission of the University of Paris Est-Créteil, the University of Turin, and by the French and Italian Ministries of Health in compliance with international laws and policies. All *in vivo* experiments were carried out with the approval of the appropriate ethical committees, and under conditions established by the European Union.

### Immunostaining of tumour sections

Frozen tumour sections were processed for immunofluorescence as previously described [37]. Sections were air-dried, fixed in zinc fixative for 10 minutes, saturated with BSA 3 %, 1 hour at RT, and incubated with either anti-ki67 (DAKO, M0722) or anti-MECA32 (rat monoclonal, BD Pharmingen 550563, clone MECA-32). After washing, sections were incubated with anti IgG Alexa Fluor-555 (Life Technology). Nuclei were stained with DAPI, and immunofluorescence images were captured by confocal microscopy. Acquisitions were carried out on multiple tissue sections (5 field per mouse, n = 5), and quantification analysis was performed with the ImageJ sofware.

### Bio-distribution assay

In order to assess the capability of N6L and N6L polyplexes to reach and distribute into the malignant lesions, a bio-distribution experiment in tumour-bearing PDAC mice was performed using green fluorescent compounds. N6L-alexa fluor 488 or N6L-alexa fluor polyplexes were injected in the tail vein, and mice were sacrificed 6 and 12 hours afterward. Pancreas, liver and kidney, as control, were excided from each mouse, and freshly included in OCT (Tissue Tek). Ten micrometer-thick sections were cut using a Leica CM1900 cryostat. Sections were air-dried, fixed in a zinc fixative (6.05g Tris, 0.35g Ca (C_2_H_3_O_2_)_2_, 2.5g Zn(C_2_H_3_O_2_)_2_, 2.5g ZnCl_2_, 3.8 mL HCl 37%) for 10 minutes, and the nuclei were counterstained with diamidino-2-phenylindole (DAPI) (Invitrogen). Stainings were analyzed by using a Leica SPEII confocal laser-scanning microscope (Leica Microsystems).

### Statistics analysis

Unless indicated otherwise, bars represent mean +/− Standard Error Mean (S.E.M.; n ≥ 3), p values have been calculated using a two-tailed or one-tailed unpaired t test using GraphPad Prism software. ^*^p < 0.05; ^**^p < 0.01; ^***^p < 0.001.

## SUPPLEMENTARY MATERIALS FIGURES



## Abbreviations

N6L: NUcleolin ANTagonist
GAG: glycoaminoglycans
DLS: dynamic light scattering
CS-C: chondroitin sulphate C
TEM: transmission electronic microscopy
PANC-1: human pancreatic carcinoma cell line
U87-MG: human glioblastoma cell line
mPDAC: murine pancreatic ductal adenocarcinoma
