# Supplementary material for: Multivalent cationic pseudopeptide polyplexes as a tool for cancer therapy
Source: Oncotarget. 2017 Sep 30;8(52):90108–22. doi: 10.18632/oncotarget.21441 (PMC5685735; doi:10.18632/oncotarget.21441)
Supplement: Supplementary file 1 [file oncotarget-08-90108-s001.pdf]

Multivalent cationic pseudopeptide polyplexes as a tool for cancer therapy

SUPPLEMENTARY MATERIALS

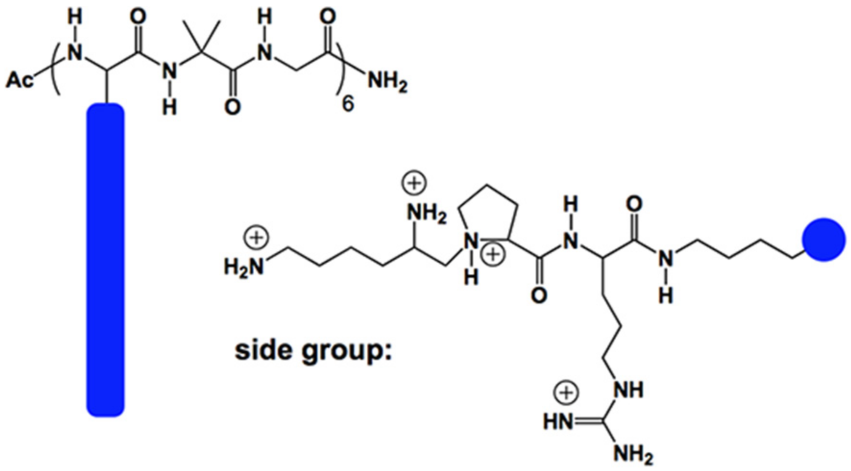

Supplementary Figure 1: Chemical structure of the N6L pseudopeptide, with the KψPR side chains portrayed as blue cylinders (counterions are acetate anions); the exact structure of the KψPR subunits is provided on the right side, with the attachment point to the backbone indicated by a blue full circle.

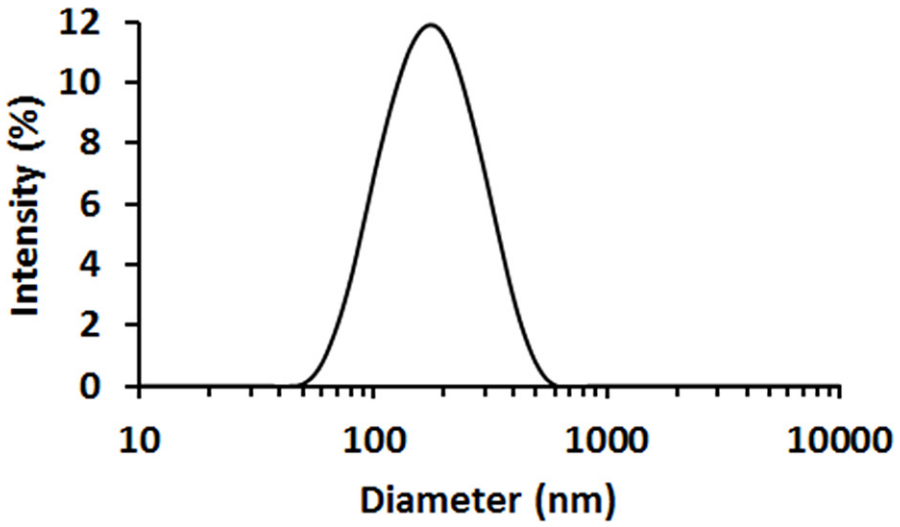

Supplementary Figure 2: Dynamic light scattering (DLS) measurements. Dynamic light scattering (DLS) measurement of particle size distribution measured after mixing heparin with N6L in water at 23°C (final concentrations of 14.7 µM and 100 µg/mL for N6L and heparin, respectively); representative results from three consecutive measurements of 15 runs each.

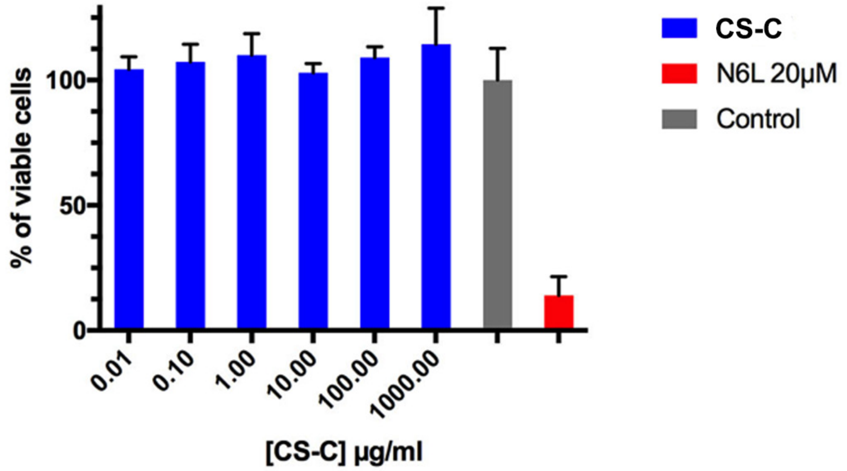

Supplementary Figure 3: Effect of CS-C on the PANC-1 viability. PANC-1 cells were treated with various concentration of CS-C ranging from 0.01 to 1000 µg/ml. After 72 hours, cell growth was quantified by AlamarBlue assay according to the recommended procedure. Histograms represent the percentage of cell growth relative to the values of untreated cells.

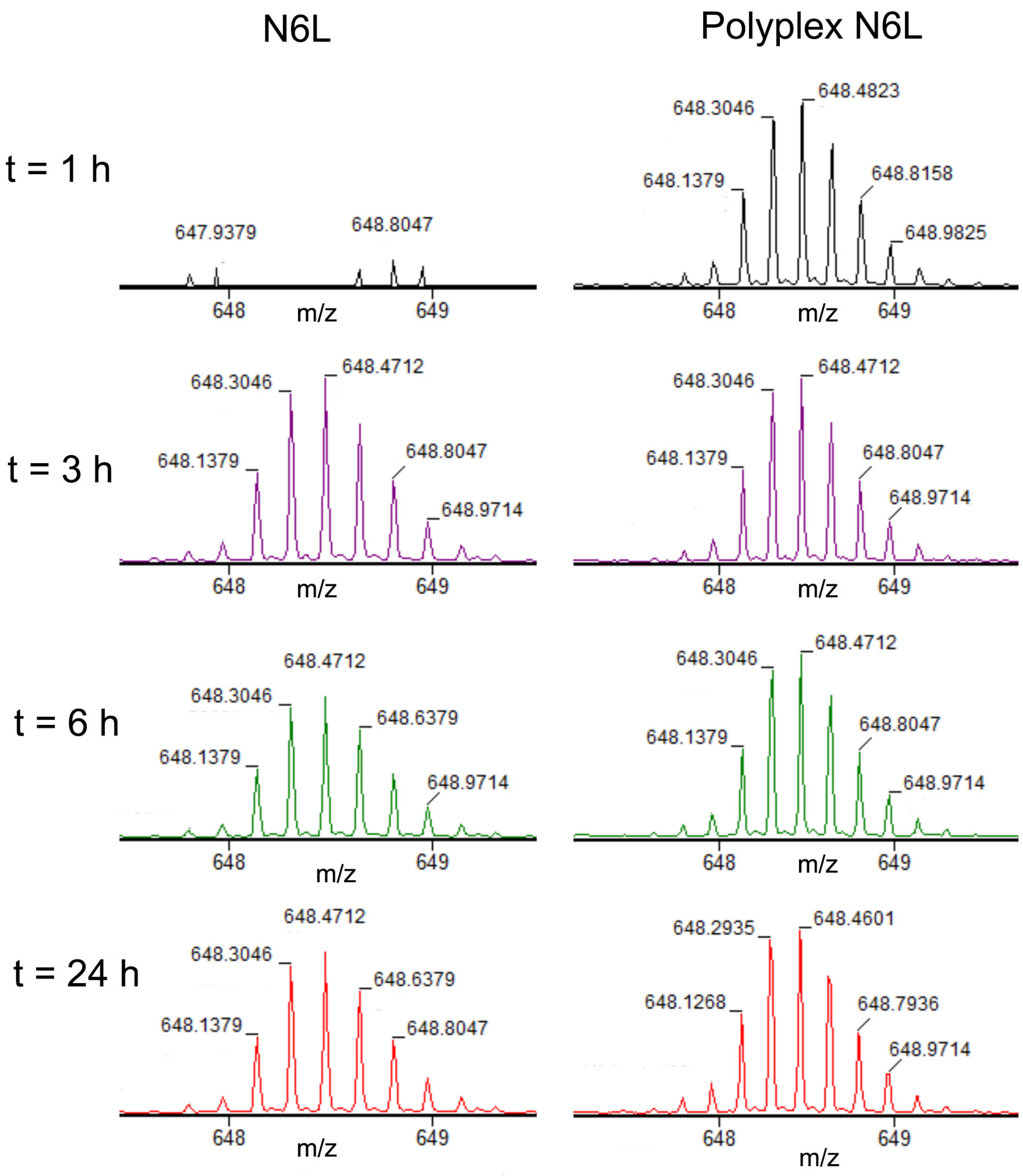

Supplementary Figure 4: MS determination of N6L presence in the dialysate. A solution of 1 mL of 73 µM N6L or of 1 mL of polyplexed N6L, with final concentrations of 73 µM and 10 µg/mL in N6L and CS-C, respectively, were independently dialyzed against 100 mL of water. 1-mL aliquots were taken at times indicated in the figure, and analysed by mass spectrometry. The peptide with m/z equal to 648.637 was identified as N6L.
